# A microbeam grazing-incidence approach to L-shell x-ray fluorescence measurements of lead concentration in bone and soft tissue phantoms

**DOI:** 10.1088/1361-6579/aaad5a

**Published:** 2018-03-29

**Authors:** Mihai Raul Gherase, Summer Al-Hamdani

**Affiliations:** California State University, Fresno, CA, United States of America

**Keywords:** x-ray fluorescence, lead, human bone, grazing-incidence XRF

## Abstract

**Objective:**

L-shell x-ray fluorescence (LXRF) is a non-invasive approach to lead (Pb) concentration measurements in the human bone. The first studies were published in the early 1980s. In the same period the K-shell x-ray fluorescence (KXRF) method using a Cd-109 radionuclide source was developed and later improved and refined. Lower sensitivity and calibration difficulties associated with the LXRF method led the KXRF to be the most adopted method for *in vivo* human bone Pb studies. In the present study a microbeam-based grazing-incidence approach to Pb LXRF measurements was investigated.

**Approach:**

The microbeam produced by an integrated x-ray tube and polycapillary x-ray lens (PXL) unit was used to excite cylindrical plaster-of-Paris (poP) bone phantoms doped with Pb in seven concentrations: 0, 8, 16, 29, 44, 59, and 74 *µ*g g^−1^. Two 1 mm- and 3 mm-thick cylindrical shell soft tissue phantoms were made out of polyoxymethylene (POM) plastic. Three bone-soft tissue phantom sets corresponding to the 0, 1, and 3 mm POM thickness values resulted. Each phantom was placed between the microbeam and the detector; its position was controlled using a positioning stage. Small steps (0.1–0.5 mm) and short 30 s x-ray spectra acquisitions were used to find the optimal phantom position according to the maximum observed Sr K*α* peak height. At the optimal geometry, five 180 s x-ray spectra were acquired for each phantom set. Calibration lines were obtained using the fitted peak heights of the two observed Pb L*α* and Pb L*β* peaks.

**Main results:**

The lowest detection limit (DL) values were (2.9 ± 0.2), (4.9 ± 0.3), and (23 ± 3) *µ*g g^−1^, respectively. The order of magnitude of the absorbed radiation dose in the POM plastic for the 180 s irradiation was estimated to be <1 mGy.

**Significance:**

The results are superior to a relatively recently published LXRF phantom study and show promise for future designs of *in vivo* LXRF measurements.

## 1. Introduction

Lead (Pb) is a well-known toxic element. While its toxicity has been known for centuries, extensive research in the past several decades has revealed the serious negative effects that various Pb exposure levels have on human health. Increasing worldwide awareness by the public, health, and governmental institutions and organizations has led to systematic Pb removal from many commonly-used chemicals such as paints and gasoline. However, occupational Pb exposure and implementation of Pb-free standards and regulations in developing countries remain important public health concerns. Also, published studies in the last two decades demonstrated correlations between developmental problems and even low Pb blood concentration (<10 µg/dL) in children (Canfield *et al* 2003, Lanphear *et al* 2005, Jusko *et al* 2008) as well as links between Pb exposure and various health problems in adults (Schaumberg *et al* 2004, Navas-Acien *et al* 2007, 2008, 2009, Shih *et al* 2007, Weisskopf *et al* 2009). While blood Pb concentration measurement remains the metric of choice for quantitative clinical assessments of individual Pb exposure, it has been known for a long time that most of the Pb in the adult human body resides in the bone (Barry and Mossman 1970, Barry 1975). Moreover, the stable Pb isotope early study of Rabinowitz *et al* (1976) estimated a biological half-life of blood Pb of about 30 d, which is much shorter than the occupational or environmental Pb exposures spanning years or decades. Therefore, *in vivo* bone Pb concentration measurements are more representative of the cumulative effects of the prolonged or continuous human Pb exposure. The history of *in vivo* bone Pb concentration measurements using the x-ray fluorescence (XRF) emission process spans four decades and the data provided by the *in vivo* bone Pb concentration measurements taken during this period significantly contributed to the knowledge of Pb kinetics in the human body during and after the Pb exposure as demonstrated in the review article by Chettle (2005).

Excitation of Pb atom innermost K-shell electrons by photoelectric absorption requires high energy x-ray photons: the K-edge is 88.006 keV (Deslattes *et al* 2003). The *in vivo* K-shell XRF (KXRF) method developed by Somervaille *et al* (1985) used the tibia bone Pb excitation by the 88.04 keV gamma-ray photons from ^109^Cd radio-nuclide (Dryak and Kovar 2006) and an internal calibration method based on the ratio between the observed Pb KXRF peaks and the 88 keV elastic scattering peak. This method, typically employing acquisition times of 30 min and a 180° backscatter geometry, was most widely adopted for *in vivo* bone Pb studies. The detection limit (DL) of Pb bone concentration measured using the KXRF method improved considerably over a span of two decades from 16–20 *µ*g Pb/g-bone mineral in the mid-1980s to around 2 *µ*g Pb/g-bone mineral using four-detector systems (Nie *et al* 2006, Fleming and Mills 2007). A recent general population survey data showed that tibia bone Pb concentration KXRF measurements varied from below the DL to as high as 20 *µ*g Pb/g-bone mineral for the tibia bone (Behinaein *et al* 2017).

The L-shell XRF (LXRF) tibia bone Pb measurement was first demonstrated in the early 1980s using an ^125^I radionuclide (Wielopolski *et al* 1983) and later using the partly polarized x-rays from an x-ray tube with a silver (Ag) target (Wielopolski *et al* 1989). The x-ray tubes have the advantages of the turn off/on option for its x-ray beam and, with the more recent development of portable x-ray spectrometers, there is also the added advantages of size and portability as well as shorter acquisitions times (Fleming *et al* 2011, Nie *et al* 2011). The original LXRF Pb measurement method developed by Wielopolski *et al* (1989) was closely scrutinized by Todd (2002a). In summary, Todd’s analysis indicated that the ultrasound soft tissue thickness measurements combined with oversimplifying assumptions regarding the soft tissue x-ray attenuation properties can lead to significant systematic uncertainties in the Pb concentration measurements. Todd also experimentally explored LXRF system optimization (Todd 2002b) using various x-ray beam polarizers and an x-ray tube with molybdenum (Mo) target. The optimal LXRF system was then used to measure Pb concentration *ex vivo* in adult human cadaver tibiae. Comparison with atomic absorption spectrometry Pb concentration measurements showed a poor correlation between the two measurement methods, particularly for intact leg Pb measurements (Todd *et al* 2002).

The Pb LXRF signal can be enhanced by optimizing the trade-off between the x-ray excitation of the 13.0 keV-bound Pb L-shell electrons and the spectral overlap between the Compton scattered peak and the Pb L*α* and Pb L*β* peaks. The optimization was the object of a recent study by Gherase *et al* (2017) using three incident photon energies (15.8, 16.6, and 17.5 keV) of a synchrotron-generated x-ray beam and two excitation-detection geometries corresponding to the 90° and 135° scattering angles. While the calibration line slope data for the bare bone phantoms clearly favored the lower 15.8 keV energy, the addition of the soft tissue phantom complicated the analysis due to the significant spectral overlap aforementioned.

In the present study, the authors explored Pb detectability in plaster-of-Paris (poP) bone phantoms using an x-ray tube integrated with a polycapillary x-ray lens (PXL) in a grazing-incidence geometry. In conventional XRF geometry, large angles (>10°) between the incident x-ray beam and sample surface are employed. In a grazingincidence XRF geometry, a small angle (<10°) between the incident x-ray beam and sample surface is typically employed to minimize the scatter background in the acquired x-ray spectra.

Various XRF and x-ray diffraction (XRD) applications using PXLs were summarized by MacDonald and Gibson (2003). Studies involving PXLs in medical imaging research such as mammography (Abreu *et al* 1995), detection of gold nanoparticles used as a contrast agent for x-ray imaging (Ricketts *et al* 2013), and optical luminescence techniques targeting small-animal imaging (Zhang *et al* 2017), were also reported. Overall, biological or medical applications involving PXLs were limited to various research labs, and, to our best knowledge, *in vivo* XRF measurements of trace elements in the human body using PXLs were not reported.

The PXL produced a highly collimated and focused x-ray beam which is herein referred to as the microbeam. There were two microbeam-related factors that motivated the study. First, the microbeam reduces the inelastic (Compton) and elastic (Rayleigh) scattering angle range of the photons reaching the detector by minimizing the volume of first-interactions. Second, the bone scatter component can be minimized by a grazing-angle approach in which the x-ray microbeam is incident only on the outermost layer of the bone. This approach was supported by the histological distribution of Pb within the human bone: a higher Pb concentration in the outer layer of the cortical bone than within its inner volume and trabecular bone (Zoeger *et al* 2005).

The poP and polyoxymethylene (POM, (CH_2_O)_*n*_) were used as phantom materials for the human cortical bone and the overlying soft tissue, respectively. Calibration lines were obtained for the bare bone, 1 mm, and 3 mm POM thickness phantom sets using the measured peak height of the observed Pb L*α* and Pb L*β* peaks. The measurements were performed at the optimal grazing-angle geometry. This geometry was determined based on the maximum observed peak height of strontium (Sr) in several short 30 s spectra acquired at different positions of the bone phantoms relative to the incident x-ray beam. Sr is a well-known contaminant of commercial poP products with estimates of its concentration at a few mg per g of calcium (Ca) (Pejović-Milić *et al* 2004). Sr was not of interest in this study and it was assumed to be uniformly distributed within the poP bone phantoms. Therefore, constant peak height measurements of the Sr K*α* and Sr K*β* for different poP bone phantoms were expected given the same XRF experimental conditions. Instead, variations of the Sr K*α* peak height measurements were observed for the seven Pb-doped samples at the optimal grazing-angle geometry. These variations were likely caused by inherent density differences between the bone phantoms due to the porous microstructure of the poP material. Further, Sr K*α* peak height measurements were used to correct the Pb L*α* and Pb L*β* peak height data. These corrections led to statistically significant improvements in the goodness-of-fit of the linear fitting procedures which determined the Pb calibration lines.

The detection limit (DL) value of Pb was calculated as the ratio between three times the null Pb concentration peak height uncertainty divided by the calibration line slope value. The lowest Pb DLs for the three overlying POM thickness values from 0 to 3 mm were 2.9 ± 0.2, 4.9 ± 0.3, and 23 ± 3 *µ*g g^−1^. While the 3 mm POM thickness DL value remains too high for *in vivo* human bone Pb measurements, the DL values for a soft tissue thickness below 2 mm are within the range of such applications. Bone Pb surveys of children are particularly attractive since their soft tissue thickness is, in general, lower than that of adults.

We did not directly measure the corresponding radiation dose. However, our approximate calculations based on the x-ray beam measurements gave a dose estimate below 1 mGy for the 180 s irradiations used in this study. The background values corresponding to the Pb LXRF peaks were also extracted from the spectra analysis. The data showed no significant background increase from the 1 mm to the 3 mm POM thickness experiments. In all, the results and discussion sections of this study indicate that further improvements of the Pb LXRF approach are still possible. Combined with viable novel calibration methods the effort may lead to future *in vivo* human Pb bone measurements using microbeam-based methods.

## 2. Methods

### 2.1. Sample preparation

Human bone phantoms were made of calcium sulphate hemihydrate ( ${\text{CaSO}}_{4}\cdot \frac{1}{2}H_{2}O$) which is also known as plaster-of-Paris (poP). The poP powder form (Sigma-Aldrich, St. Louis, MO) was mixed with distilled water and doped with known quantities of lead (Pb) using pipette-measured volumes of Pb standard atomic absorption solution (Sigma-Aldrich, St. Louis, MO). The precise chemical form of Pb was not known. The solvent was a diluted water-based nitric acid (HNO_3_) solution (2% w/w). The viscous mixture was then poured into aluminum cylindrical molds. The bone phantoms were rigid cylinders with a 29 mm diameter following solidification. Their masses were measured and the final Pb concentration was calculated based on the initial Pb standard solution volume measurements. The resulting poP density for each phantom was also calculated based on measured masses and volumes. The poP density value and its uncertainty provided in table 1 were calculated as the average and standard deviation of the seven bone phantoms, respectively. Seven bone phantoms were made with varying Pb concentrations of 0, 8, 16, 29, 44, 59, and 74 *µ*g g^−1^.

Soft tissue phantoms were made of polyoxymethylene (POM, (CH_2_O)_*n*_). POM density and its uncertainty were calculated based on volume water displacement and mass measurements. Relevant properties of the poP and POM phantom materials are summarized in table 1. X-ray linear attenuation coefficients were calculated based on the material mass density and elemental composition and using the x-ray mass attenuation coefficients available through the XCOM (Berger *et al* 2010) online database of the National Institute of Standards and Technology (NIST). Two cylindrical shell-shaped phantoms with a 30 mm inner diameter and 1 and 3 mm shell thickness, respectively, were machined out of a larger diameter solid cylindrical rod. To mimic *in vivo* LXRF bone Pb measurements, cylindrical bone phantoms were inserted in the POM soft tissue phantoms. The combination resulted in three phantoms sets.

During the measurements the air gaps between the bone and soft tissue phantoms due to slightly mismatched phantom diameters were eliminated by placing folded paper on the opposite side of the x-ray beam incidence. A larger diameter solid cylindrical base of the POM phantoms secured mechanical stability of the bone-soft tissue phantom assembly.

### 2.2. Experimental setup

**Figure 1:**
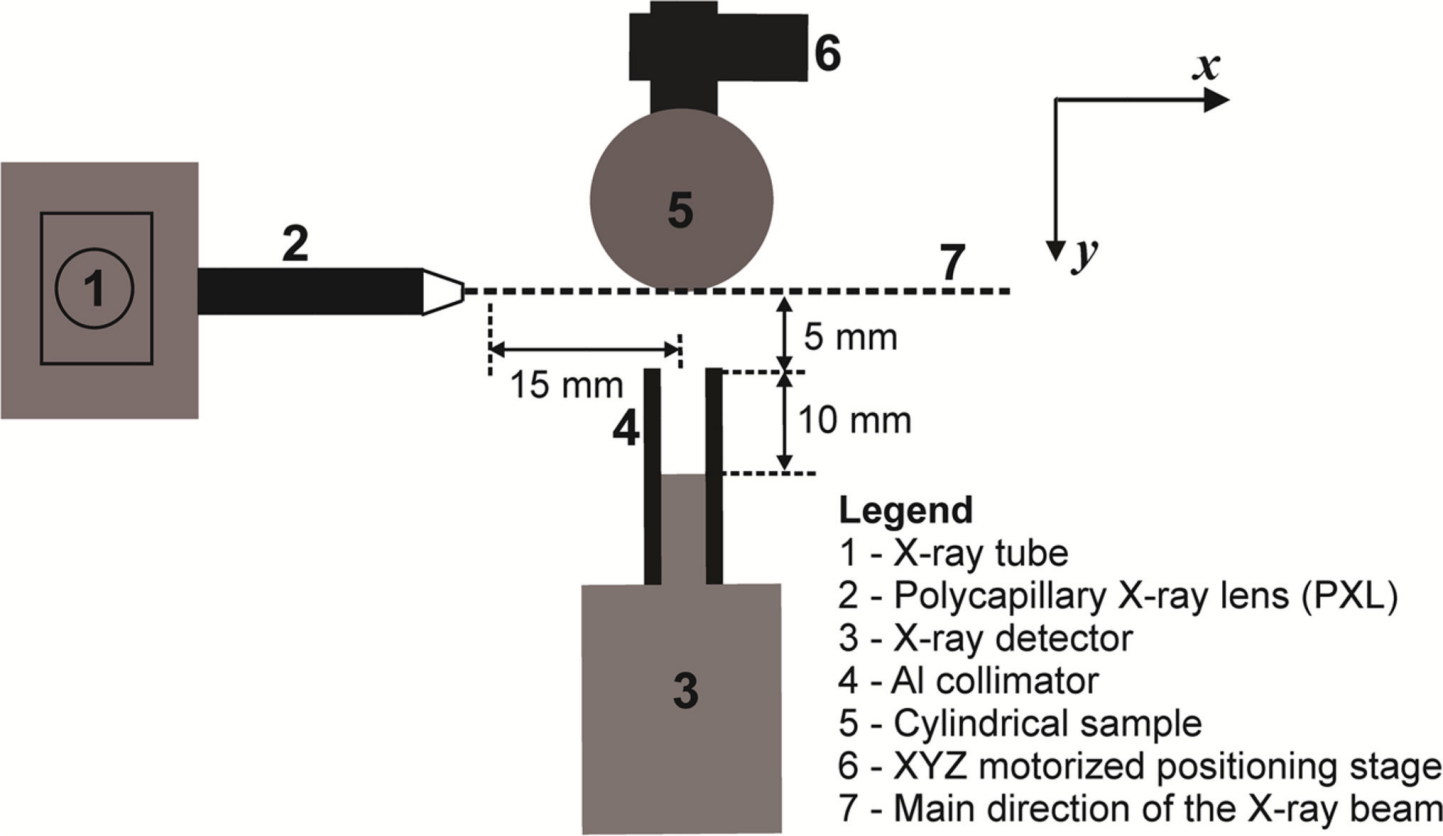
Top view schematic of the experimental setup used in the Pb LXRF measurements.

The schematic of the experimental setup used in the Pb LXRF measurements is shown in figure 1. In the figure, cylindrical sample refers to one of the three bone/soft tissue phantom combinations. The sample excitation was achieved using the microbeam produced by an integrated x-ray tube and polycapillary x-ray lens (PXL) (Polycapillary X-beam Powerflux model, X-ray Optical Systems, Inc., East Greenbush, NY, US).

**Figure 2:**
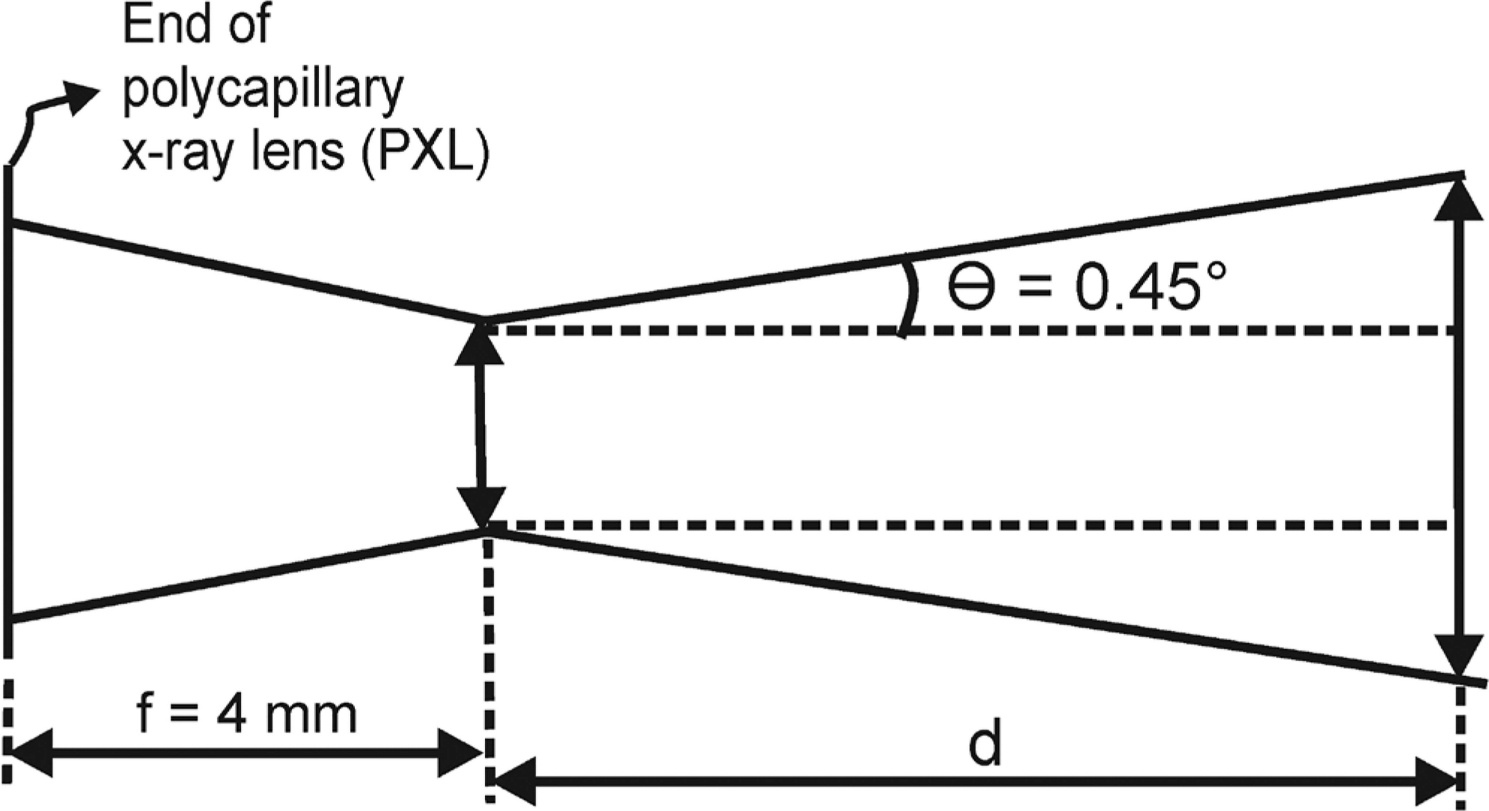
Schematic of the PXL x-ray beam geometry.

The x-ray tube had a tungsten (W) target and the x-ray lens was 10 cm in length and 1 cm outer diameter. The x-ray tube voltage and current could be varied in 0.1 kV and 1 *µ*A increments, respectively. Their maximum values of 50 kV and 1 mA were used during the LXRF measurements. Knife-edge x-ray beam size measurements performed recently in our lab determined that the x-ray lens had a focal length of 4 mm where the x-ray beam had a 24 *µ*m lateral size measured as full width at half-maximum (FWHM) at the 10 keV photon energy (Gherase and Vargas 2017). At the 10 keV photon energy, the x-ray beam divergence at a distance larger than the 4 mm focal length was measured to be 7.8 mrad (or 0.45°) as shown in figure 2. Hence, an approximate FWHM value of the x-ray beam in mm at a distance *d* from the PXL in mm can be calculated from the beam geometry of figure 2 using the following equation:

(1) $$\text{FWHM}(\text{mm})=0.024+2\times (d(\text{mm})-4)\times 7.8\times 10^{-3}.$$

It is important to note that the geometrical characteristics of the x-ray beams formed by PXLs (lateral size, focal length, beam divergence) are all dependent on the photon energy (Sun and Ding 2005). Upstream of the PXL the x-ray beam was filtered using an eight-slot wheel in which custom-made filters could be placed. A 1.8 mm-thick aluminum (Al) filter was used to attenuate the W L-shell XRF emissions in the 8–12 keV energy range. This step reduced, but did not eliminate the spectral background in this energy range. X-ray photon counting and energy measurements (i.e. x-ray spectra acquisition) were accomplished using a silicon-drift x-ray detector with integrated pulse-height analyzer (X-123 SDD, Amptek, Bedford, MA, US). The circular active area of the detector was 25 mm^2^ (or 5.6 mm diameter) and 0.5 mm thickness and the window was a 12.7 *µ*m-thickness beryllium (Be) sheet. The counting rate capability of the detector provided by the manufacturer was 10^5^ counts s^−1^.

The samples were placed on a T-shaped Al plate securely mounted on an automated XYZ modular motorized linear positioning stage assembly (Newport, Irvine, CA, US). Sample positions in the x-ray beam with 1 *µ*m accuracy were achieved with this system. The entire XRF setup was placed on an x-ray shield consisting of a 56 × 62 cm^2^ and 6.35 mm-thickness Al plate. The plate was covered by a stainless steel box with a slightly smaller base area and 46 cm in height which could be opened to allow operating access to the XRF setup. The cover could be manually opened when the x-ray beam was turned off. The integrated x-ray tube and PXL unit was mounted in a fixed position inside the x-ray shield while the x-ray detector and the positioning stage could be securely positioned anywhere around the x-ray beam direction using a magnetic base holder and magnetic strips, respectively. The integrated x-ray tube and PXL unit was also equipped with an electromechanical beam shutter and a green and yellow light beacon. When the green light turned on, it indicated that the power supply was on and that the beam shutter was closed. When the yellow light turned on, it indicated that the power supply ramped to the voltage and current values selected by the user. When the yellow light was on and green light turned off, it indicated that the shutter was open and the x-ray shield lid should be closed to avoid radiation exposure of nearby users. Two L-shaped metallic pipes guided the connecting cables with the x-ray tube power unit and the laptop computer which was used to operate all devices. The entire x-ray shield and XRF assembly was mounted on an optical table (Newport, Irving, CA, US) which also served as mechanical support for the x-ray tube power supply and the laptop computer.

### 2.3. LXRF Pb measurements

Based on the bone/soft tissue phantom combinations three different sets of LXRF measurements were performed: (1) bare bone phantoms, (2) bare bone and 1 mm thickness soft tissue phantom, (3) bare bone and 3 mm thickness soft tissue phantom. For each combination, all seven Pb-doped bone phantoms were used. For brevity, identifying labels were used throughout this article as summarized in table 2. Five 180 s trials were acquired for each Pb concentration. The dead time indicated by the x-ray detector software interface was less than 1% for each measurement. For each trial, the peak height values of the two observed Pb L*α* and Pb L*β* peaks were measured. In all, six calibration lines (peak height versus Pb concentration) resulted. The sample was positioned just outside the x-ray beam on the positioning stage. The middle of the PXL circular end—as seen from the direction of the x-ray beam—was used as an approximate visual guide to the location of the x-ray beam.

Using the positioning stage, the sample was then moved towards the x-ray beam in small steps in the 0.1– 0.5 mm range. Smaller 0.1 mm steps were used for the bb phantom measurements while the larger 0.5 mm steps were used for the bb-3 mm phantoms. At each location, a single 30 s x-ray spectrum was acquired and the optimal position was then chosen based on the maximum observed peak height of the strontium (Sr) K*α* peak.

### 2.4. X-ray beam measurements

An approximate order of magnitude dose calculation was performed based on the measured photon fluence rate for each energy. The experimental setup shown in figure 2 was re-arranged such that the x-ray detector window faced the PXL with a 5 cm distance between the two. At this distance the x-ray beam lateral size calculated using equation (1) was ~0.8 mm which was well within the dimensions of the detector. In order to mitigate dead time counting losses of the detector at levels below 1%, the x-ray tube current was set at 2 *µ*A which was 50 times lower than the 1 mA current used in the LXRF measurements.

### 2.5. Data analysis

The raw x-ray spectra were analyzed using OriginPro 2015 (OriginLab Northampton, MA, US) statistical data analysis and plotting software. The Pb L*α* and Pb L*β* peaks were fitted separately by selecting only the data around the peaks. The peaks were modeled as a Gaussian function with a linear background according to the following equation:

(2) $$y=a+\text{bx}+H\text{ exp}[-\frac{{\left(x-x_{0}\right)}^{2}}{2\sigma^{2}}].$$

In equation (2), variables *y* and *x* represent the number of counts and photon energy, respectively. The variables *a* and *b* are the parameters of the linearly-modelled background and *H, x*_0_, and *σ* are the peak height, center, and standard deviation parameters of the Gaussian peak model, respectively. The observed Sr K*α* and Sr K*β* peaks were fitted simultaneously using a model as in equation (2) to which a second Gaussian function was added. Nonlinear curve fitting component of the OriginPro software based on the Levenberg–Marquardt numerical minimization algorithm was employed to fit the data to equation (2) model. For each channel with *N* counts, the standard deviation of the measurement was taken to be equal to $\sqrt{N}$ according to the Poisson statistics. Thus, the corresponding statistical weight was equal to 1/*N*. Goodness-of-fit was simultaneously monitored using the following built-in options of the OriginPro software: (i) reduced chi-square value (*χ*^2^/*n*), (ii) the coefficient of determination (R^2^), and (iii) visualization of the fitted function and data plots. In general, all parameters in equation (2) were treated as free parameters in the fitting procedures. However, for low Pb concentrations near the detection limit, the Pb L*α* and Pb L*β* peaks corresponding to the bone and soft tissue phantom measurements were hardly distinguishable from the background, and, therefore, the peak fitting routine was aided by assigning the center (*x*_0_) and standard deviation (σ) parameters with the measured values of the well-resolved Pb peaks from the bare bone phantom LXRF measurements. The same approach was employed for the null Pb concentration spectra analysis.

The final results were calculated as the mean and the standard deviation of the mean (SDOM) of the five fitted peak height values corresponding to the 180 s trials. These calculations and organization of the final results were performed using Microsoft Office Excel software (Microsoft, Redmond, WA, US). Sample and final results plots were also done using the OriginPro software. X-ray atomic transitions of the observed XRF peaks were identified using the tabulated data from Deslattes *et al* (2003). The Sr K*α* peak height in identical experimental conditions should be constant since Sr was a homogeneous contaminant of the plaster-of-Paris compound. The observed variations of the Sr K*α* peak height data indicated that there were systematic uncertainties in the measurement procedure. These were likely caused by density differences amongst the bone phantoms due to the porous nature of the poP material. This observation led to corrections of the Pb L*α* and Pb L*β* peak height data. The Pb peak height values denoted generic by *H*_Pb_ were corrected using the corresponding Sr K*α* peak height denoted by *H*_Sr_ and its maximum value *H*_Sr,max_ out of the set of seven Pb concentration measurements as follows:

(3) $$H_{\text{Pb},\text{corr}}=H_{\text{Pb}}\frac{H_{\text{Sr},\text{max}}}{H_{\text{Sr}}}.$$

From the calibration lines of the form *y = y*_0_ + *sx* obtained with the Pb L*α* and Pb L*β* peak height data, the DL of the Pb concentration measurement was calculated using the following equation (Fleming *et al* 2011):

(4) $$\text{DL}=\frac{3\sigma_{0}}{s}.$$

In equation (4) the *σ*_0_ represents the SDOM obtained from the five trials of the null Pb concentration bone phantom and *s* represents the slope of the calibration line. Using error propagation of statistically-independent variables, an uncertainty on DL, *σ*_DL_, can be calculated using the slope, *s*, and the error on the slope, *σ*_s_, as follows:

(5) $$\sigma_{\text{DL}}=\text{DL}\frac{\sigma_{s}}{s}.$$

## 3. Results

### 3.1. Pb LXRF measurements

**Figure 3:**
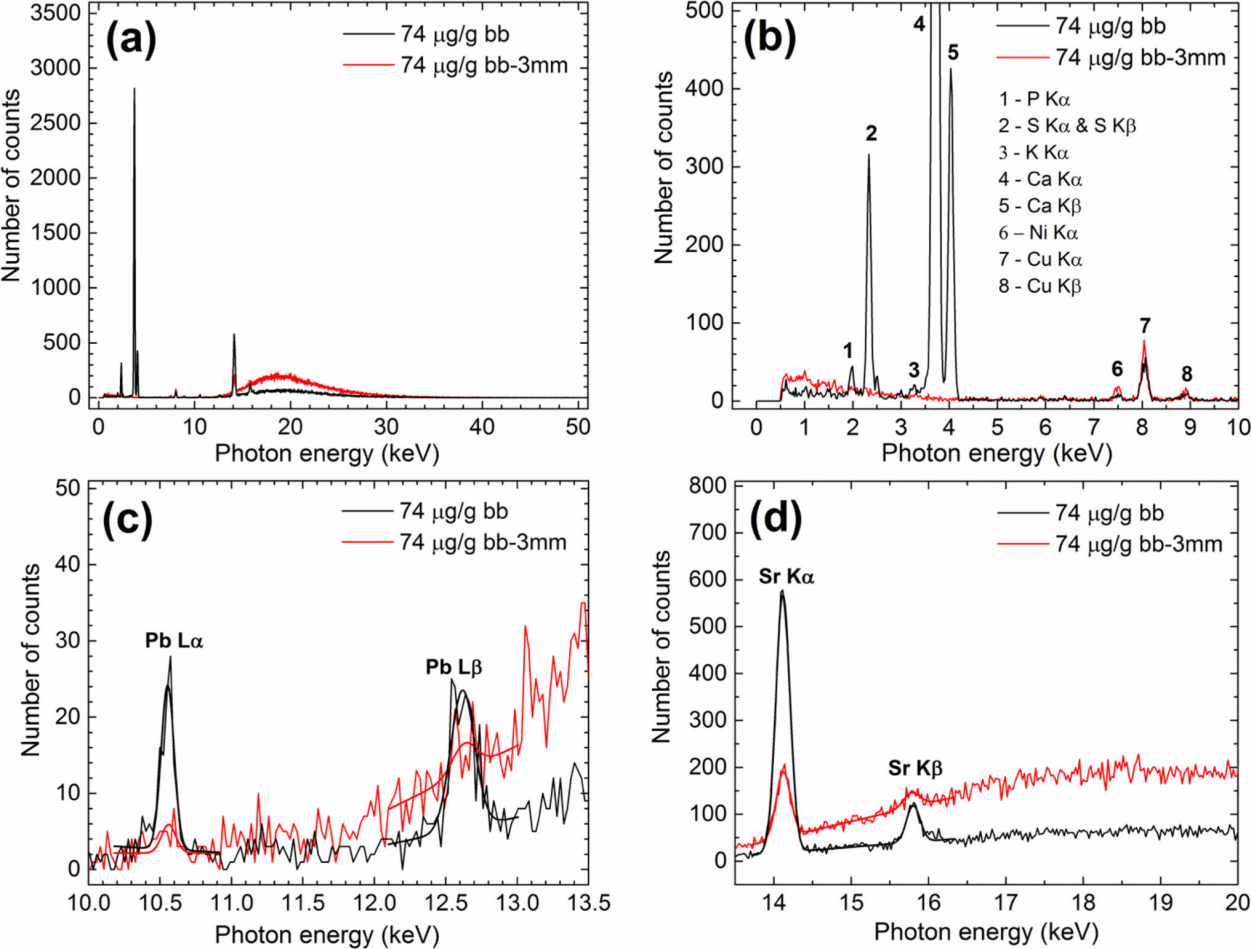
Two sample x-ray spectra corresponding to the 74 *µ*g g^−1^ Pb concentration of the bb (black line) and bb-3 mm (red line) LXRF measurements. The four plots focus on different energy ranges: (a) 0–50 keV; (b) 0–10 keV energy range; (c) 10–13.5 keV (Pb LXRF peaks and Gaussian peak fitting); (d) 13–20 keV (Sr K*α* and Sr K*β* peaks and Gaussian fitting of the peaks).

Sample plots of the x-ray spectra obtained in the Pb LXRF experiments are shown in figure 3.

In plot (a) the larger scattered bremsstrahlung wide peak in the bb-3 mm spectrum can be noticed due to the increased number of scatter events in the soft tissue phantom. A larger number of XRF peaks can be observed in plot (b) in the bb spectrum in the absence of the x-ray attenuation within the POM soft tissue phantom.

**Figure 4:**
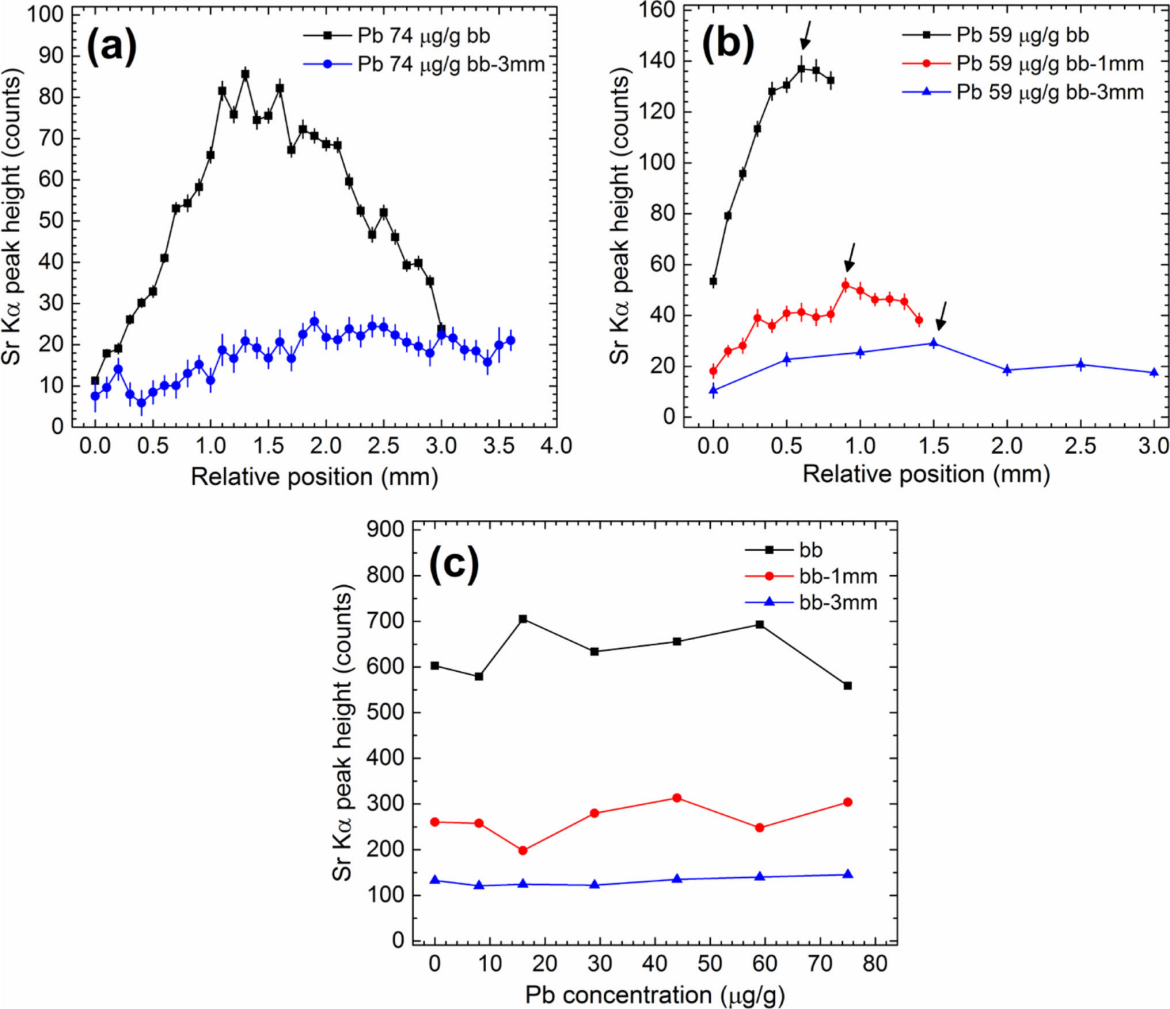
(a) Plot of the Sr K*α* peak height variations when the position of the phantoms is varied in small 0.1 mm steps in the *y*-axis direction shown in figure 1. The 74 *µ*g g^−1^ Pb concentration of the bb and bb-3 mm phantom assembly were used. (b) Sample plots of the Sr K*α* peak height variations obtained using 30 s x-ray spectra acquisitions. The optimal phantom positions indicated by arrows correspond to the maximum values of these curves. (c) Sr K*α* peak height values calculated as the mean of the five 180 s trials for each phantom assembly and Pb concentration. The error bars were calculated as standard deviation of the mean and were too small to be distinguished from the data points.

Figure 4 shows variations of the Sr K*α* (14.1 keV) peak height in two distinct situations: (i) position of the phantom relative to the x-ray beam was varied in 0.1 mm and 0.5 mm steps (plots (a) and (b)); and (ii) x-ray spectra were acquired to produce the Pb L*α* and Pb L*β* calibration lines at the optimal positions of the three phantom sets (plot (c)). In figure 4(b), sample plots of the Sr K*α* data which determined the optimal phantom position are shown. It can be seen that optimal phantom position was achieved roughly within the 1, 1.5, and 3 mm distances corresponding to the bb, bb-1 mm, and bb-3 mm phantom measurements, respectively. The increasing trend was related to the experimental approach in which the thickness of the POM soft tissue phantom was not accounted for in the initial phantom positioning procedure. The Sr K*α* peak height data shown in the plots from figure 4(c) were used to perform Pb L*α* and Pb L*β* peak height corrections using equation (3) as described in section 2.5.

**Figure 5:**
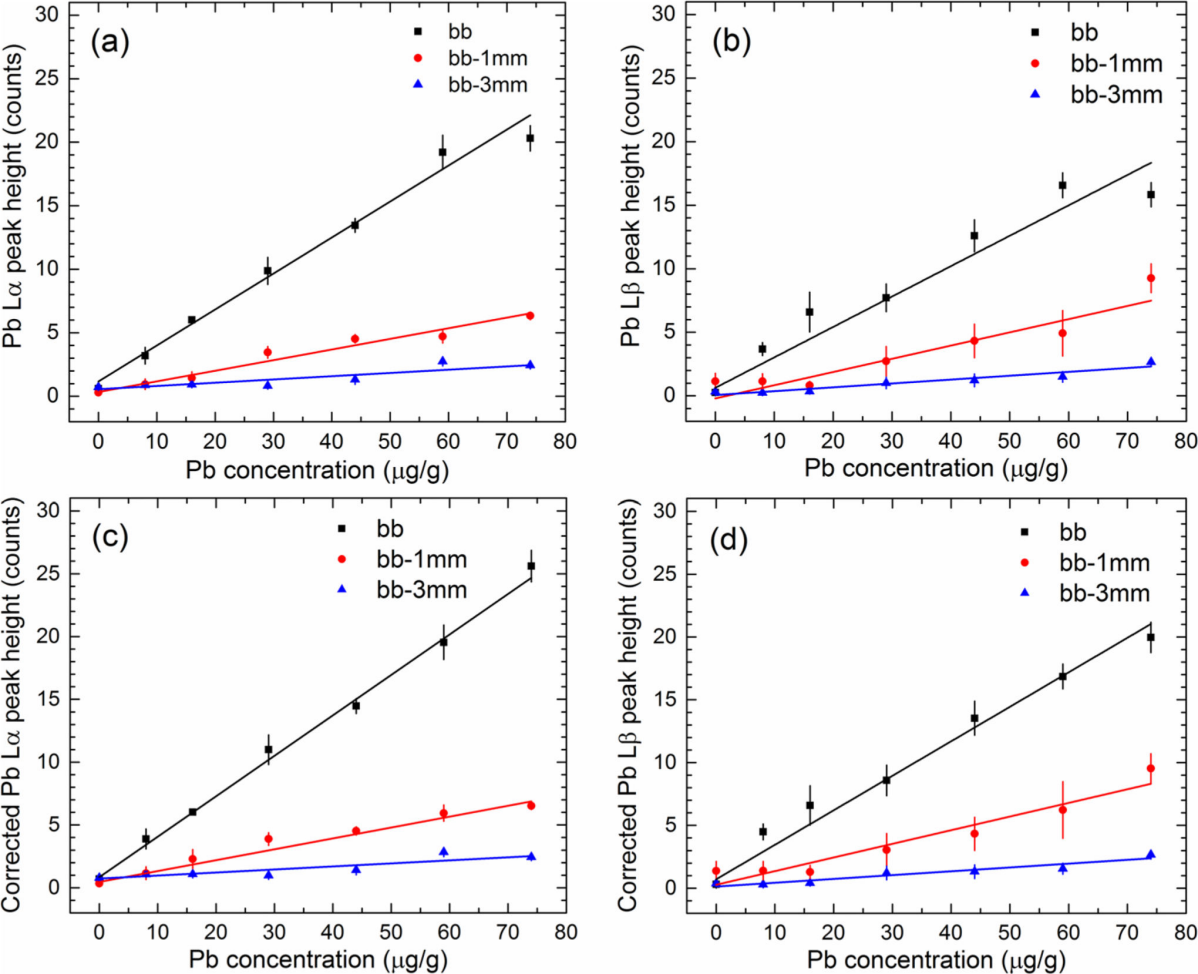
Raw (panel plots (a) and (b)) and corrected (panel plots (c) and (d)) calibration lines of Pb measurements based on the Pb L*α* (panel plots (a) and (c)) and Pb L*β* (panel plots (b) and (d)) peak height measurements.

The Pb LXRF measurements led to the raw and corrected calibration lines shown in figure 5. The slope and *y*-axis intercept parameter values and their standard deviations obtained from the linear fitting routines are shown in table 3. The corresponding reduced chi-square values for each calibration line are also provided in table 3. A visual inspection of the plots in figure 5 and the reduced chi-square values from table 3 show that the equation (3) corrections improved the calibration line, particularly for the bb measurements. The *p* value column in table 3 indicates if the observed chi-square value departure from the expected unity value is statistically significant at the 5% level. The *p* values for the corresponding *d* = 7 − 2 = 5 degrees of freedom chi-square probability distribution function were compared to the 5% level using the statistical tables from the Taylor textbook (Taylor 1997).

The uncertainty in the null Pb concentration phantom trials and the values of the MDL and its uncertainty calculated using equations (4) and (5) are provided in table 4.

### 3.2. X-ray beam measurements and absorbed dose calculations

**Figure 6:**
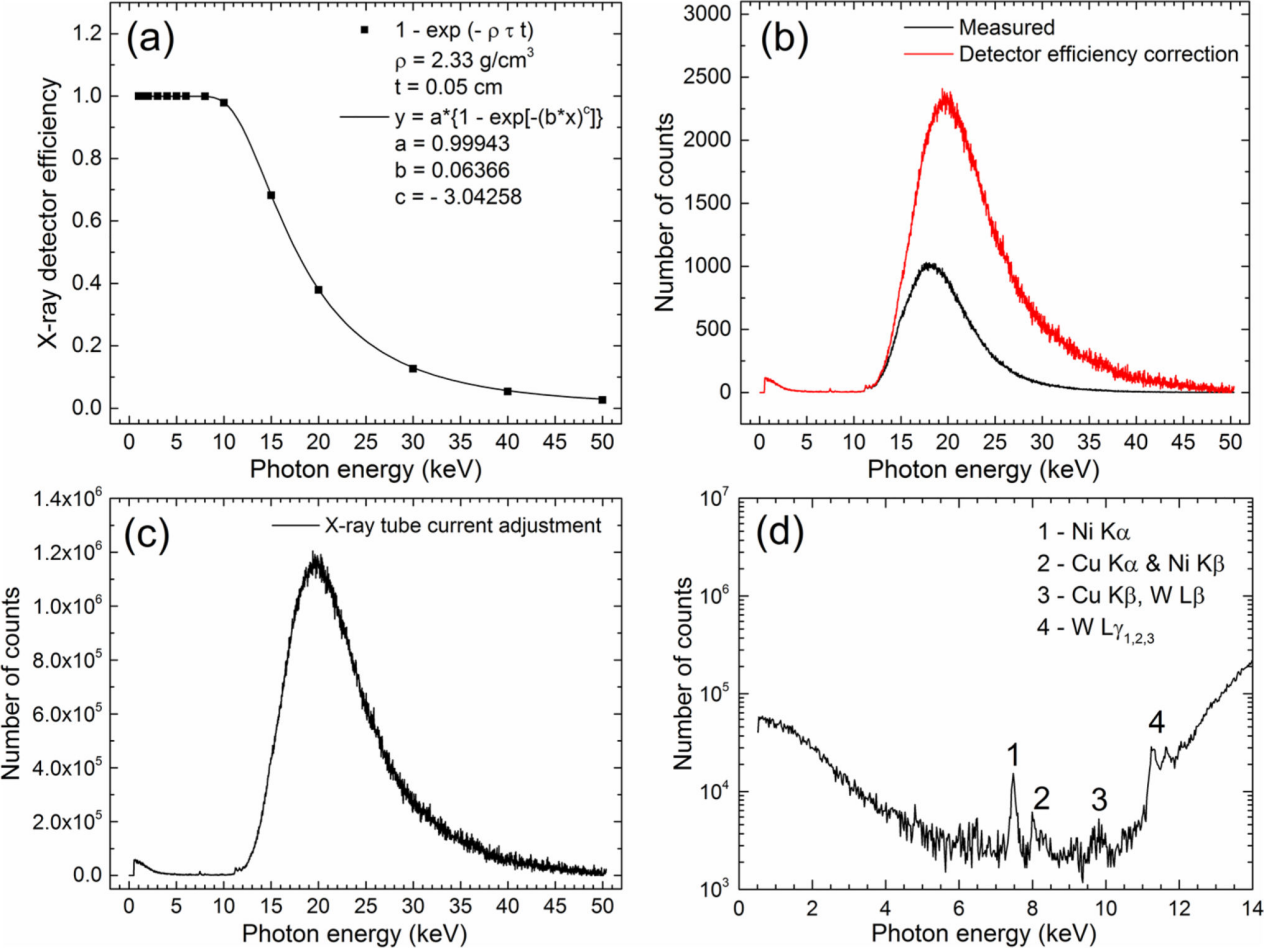
(a) Plot of the 0.5 mm thick Si x-ray detector efficiency expressed as the photoelectric absorption factor 1 − exp(*−ρτt*) where *ρ* and *t* are the Si mass density and thickness and the *τ* is the photoelectric absorption part of the mass attenuation coefficient of Si taken from the XCOM database. (b) Plots of the measured and detector-efficiency-corrected x-ray spectra. (c) Plot of the x-ray spectrum adjusted for the 1 mA x-ray tube current used in all Pb LXRF measurements. (d) Semilogarithmic plot of the corrected x-ray spectrum in the 0–14 keV energy range.

The results of the x-ray beam measurements and subsequent corrections are summarized in the plots of figure 6. The count rate calculated from the raw spectrum data (black line in the figure 6(b) plot) was 1.3 × 10^3^ counts s^−1^, well within the 10^5^ counts s^−1^ processing rate of the x-ray detector. The measured x-ray spectra were corrected for the detection efficiency of the 0.5 mm Si layer and were also adjusted from the 2 *µ*A tube current used during x-ray beam measurements to 1 mA x-ray tube current used during Pb LXRF measurements. An x-ray beam rate of ~ 2 × 10^6^ photons s^−1^ was calculated by summing all corrected counts shown in figure 6(c) and dividing by the 300 s acquisition time.

The XRF peaks shown in figure 6(d) plot indicate the presence of Ni, Cu, and W elements. The first two were identified as contaminants of the Al collimator, while W lines originated from the x-ray tube W target material.

A rough estimate of the absorbed dose was calculated employing a number of simplifying assumptions. First, the absorbed dose in the poP bone phantom material was neglected. The energy *E*_beam_ carried by the x-ray beam in the 3 min (or 180 s) time of the Pb LXRF measurement trial was the sum of *N_i_* counts of energy *E_i_* over all 2048 channels:

(6) $$E_{\text{beam}}=\frac{180}{300}\sum_{i=1}^{N}N_{i}E_{i}.$$

For each photon energy *E_i_* the fraction of absorbed energy *f_i_* along path length *L* = 1.11 cm for bb-1 mm and *L* = 1.99 cm for the bb-3 mm in the POM soft tissue material was calculated using the following approximation:

(7) $$f_{i}\approx 1-\text{exp}[-\rho \mu (E_{i})L].$$

In equation (7) *ρ* = 1.42 g cm^−3^ was the density of the POM material also provided in table 1 and *µ* (*E_i_*) is the mass attenuation coefficient of the POM material corresponding to the photoelectric and Compton interactions and was calculated using the XCOM tables.

This is an approximation, since not all Compton-interacting photons deposit their entire energy within the volume of interest. Hence, the approximate average dose *D* was calculated as the absorbed energy *E*_abs_ divided by the POM mass *m*:

(8) $$D\overset{\text{def}}{=}\frac{E_{\text{abs}}}{m}\approx \frac{\frac{180}{300}\sum_{i=1}^{N}N_{i}E_{i}f_{i}}{\rho L\pi r^{2}}.$$

In equation (8) it was assumed the energy absorption occurred within the small x-ray beam volume. The x-ray beam radius *r* = FWHM/2 = 0.137mm was calculated using equation (1) at distance *d* = 20mm. For bb-1 mm and bb-3 mm 180 s trials the absorbed dose was estimated at 0.7 and 0.5 mGy, respectively.

## 4. Discussion

### 4.1. Pb LXRF measurements

The data plots of figures 4(a) and (b) can be explained by the x-ray attenuation of the incident beam and that of the emergent Sr K*α* photons as the phantom is incrementally positioned towards the x-ray beam. The data in plot 4(c) can be explained by several possible factors. (1) poP bone phantoms had microscopic air pockets and surface irregularities on the submillimeter scale similar to that of the x-ray beam which led to a different XRF experiment for each sample despite the fact that Sr could be considered uniformly distributed throughout the bone phantom. (2) Optimal geometry was perhaps not achieved equally well for all samples given that the initial sample positioning was only determined visually and not determined in a reproducible manner. (3) The Sr K*α* peak height variation appears to decrease with increasing thickness of the POM soft tissue phantom as shown plot (c). This is also related to the much broader maximum noticed in the bb-3 mm experiment compared to that of the bb measurements shown in the plots from figure 4(a). The systematic uncertainties noted for the Sr K*α* peak data were very likely responsible for the larger-than-unity *χ*^2^/*n* values of the bb calibration line shown in table 3. Ad hoc corrections given by equation (3) reduced these values and improved the linearity as can be seen in the panel plots (c) and (d) of figure 5. Sr and Pb have different XRF photon energies and photoelectric absorption cross sections. There were also differences in the x-ray attenuation of the incident and emergent XRF photons. This means an optimal XRF detection of Sr was not entirely optimal for Pb detection. A useful test of reproducibility in optimal positioning would have been to perform the LXRF measurements with the same Pb concentration phantom several times starting from the positioning stage of the experiment, and not just mere automated repetitions of the x-ray spectra acquisition. Due to the relatively long experimental effort required, as well as the improvement demonstrated by the Sr K*α* correction for the bb calibration line measurements, this test was not performed. The results of this study and the present discussion indicate the utility of such effort in future investigations using a similar microbeam technique.

An important part of improving Pb LXRF measurement is the slope of the calibration line which is an important component of the sensitivity of any applied XRF method. The slope values, *s*, for each phantom set of Pb LXRF measurements are provided in table 3. The observed decrease in its value with the increasing of the overlying POM thickness denoted by *t* was expected and was due to the exponential attenuation of the incident and emergent x-ray photons. Let us assume that incident x-ray photons of energy *E_i_* cross a thickness *t_i_* of the POM soft tissue phantom to reach the bone and the linear attenuation coefficient of POM at this x-ray photon energy is *µ_i_*. Also, the emergent Pb L*α* and Pb L*β* photons have linear attenuation coefficients *µ_α_* and *µ_β_* and cross a POM soft tissue phantom layer of thickness *t*. Then, the relationship between the Pb L*α* or Pb L*β* slope of the bb phantom, *s_α,β_* (0), and that of a bb and soft tissue layer of thickness *t, s_α,β_* (*t*) is

(9) $$s_{\alpha ,\beta }(t)≅s_{\alpha ,\beta }(0)\text{exp}(-\mu_{i}t_{i}-\mu_{\alpha ,\beta }t).$$

**Figure 7:**
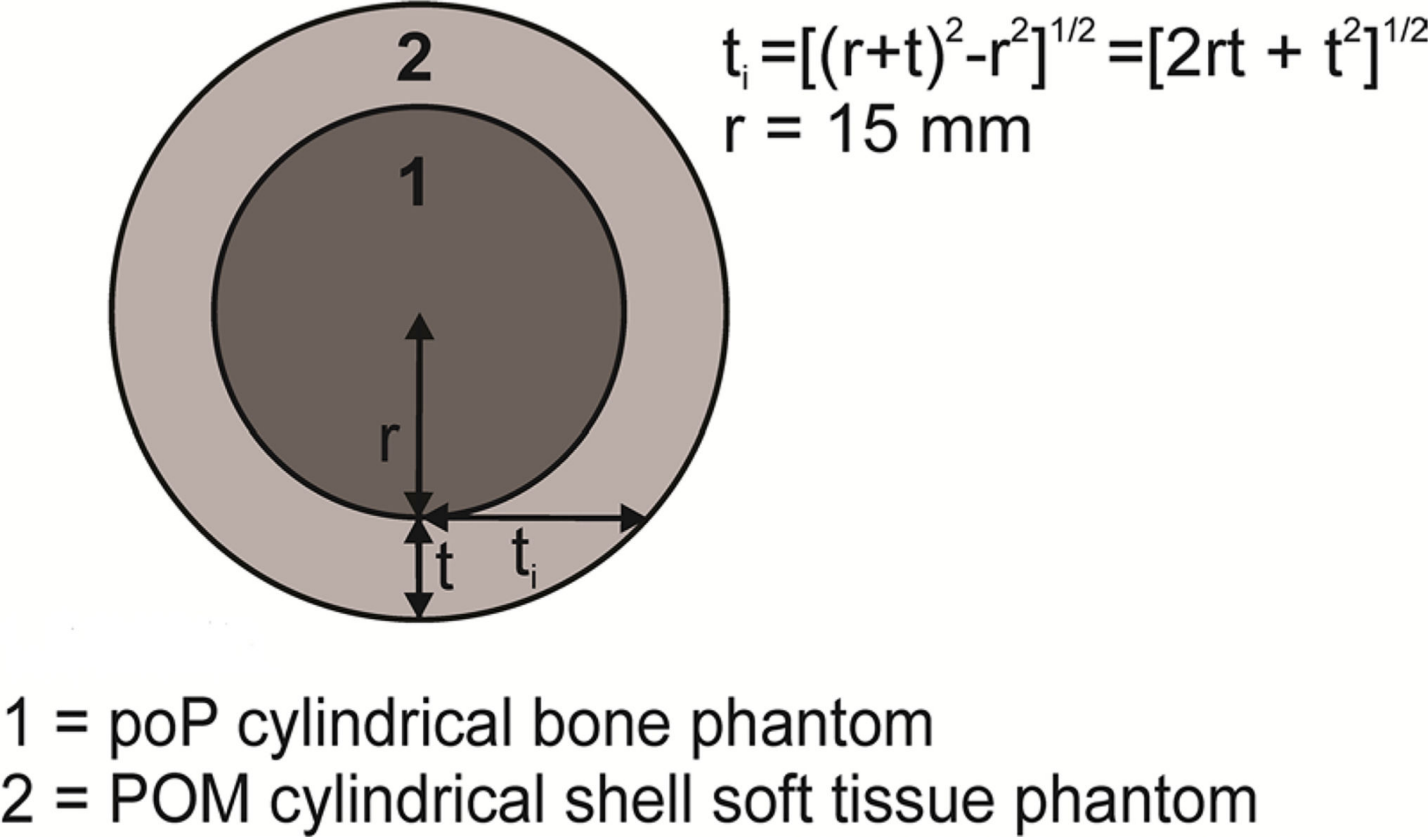
Geometry of the Pb LXRF measurements.

Equation (9) assumes well-defined paths of monoenergetic photons. While the direction of the incident microbeam and the Pb LXRF photon energies support this assumption, the incident photon energies of the microbeam and the paths of emergent photons are shaped by the physics of the x-ray tube and the transmission properties of the PXL, and the solid angle of the detector, respectively. Equation (9) also assumes the same x-ray attenuation in the poP bone phantom material for all three phantom sets, which is equivalent to having identical grazing-incidence conditions. Figure 7 shows the relationship between the soft tissue thickness values *t_i_* and *t* in the Pb LXRF measurements. The selected 0.181 mm^−1^ x-ray linear attenuation coefficient of the 15.2 keV photons corresponds to the Pb L_3_ edge energy.

**Figure 8:**
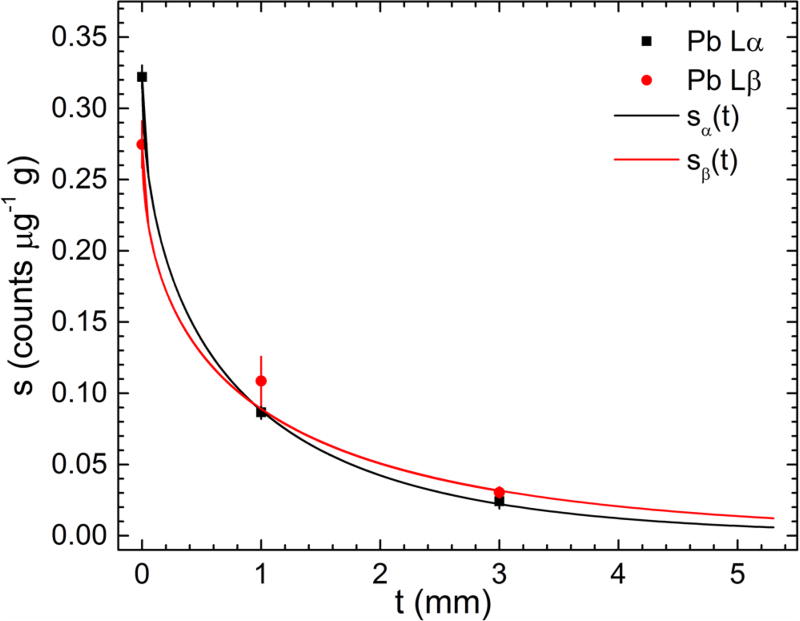
Calibration line slope data and approximate x-ray attenuation model fitted curves for the Pb L*α* (black) and Pb L*β* (red).

Figure 8 shows the calibration line slope data from table 3 and the single-parameter attenuation model fitted curves. The observed relationship for bb calibration lines *s_α_* (0) > *s_β_* (0) can be explained by the measured *L_β_/L_α_* = 0.894 for Pb (Garg *et al* 1984). The *L_β_/L_α_* ratio was larger than unity for the bb-3 mm calibration lines due to the lower x-ray attenuation of the higher energy Pb L*β* photons. The x-ray linear attenuation coefficients of the 15.2 keV incident monoenergetic photons and the Pb L*α* and Pb L*β* photons for both poP and POM materials are provided in table 5. The fitting results are shown in table 6. The two *a* parameter values from the fourth column of table 6 represent the effective x-ray linear attenuation coefficient of the POM plastic material for the Pb L*α* and Pb L*β* energies.

These values are lower than the 0.511 mm^−1^ and 0.303 mm^−1^, respectively as provided in the last row of table 5. The discrepancy has two plausible explanations: (i) the effective path length of emergent Pb LXRF photons is larger than the soft tissue phantom thickness *t*, and (ii) the x-ray attenuation in the poP bone phantom was not the same amongst all measurements. Although the simplified attenuation model did not include all details of the experimental conditions, it is worth mentioning that the data from figure 8 did not fit well an exponential decay function in which the exponent was a linear function in *t*.

The agreement between the slope data and the simplified x-ray attenuation model brings about the discussion of the scatter background. The peak fitting routine modeled the spectral background underneath the observed peaks over a narrow energy range. Hence, the relevant background data for the Pb LXRF measurements were readily available in the results of the peak fitting routines.

**Figure 9:**
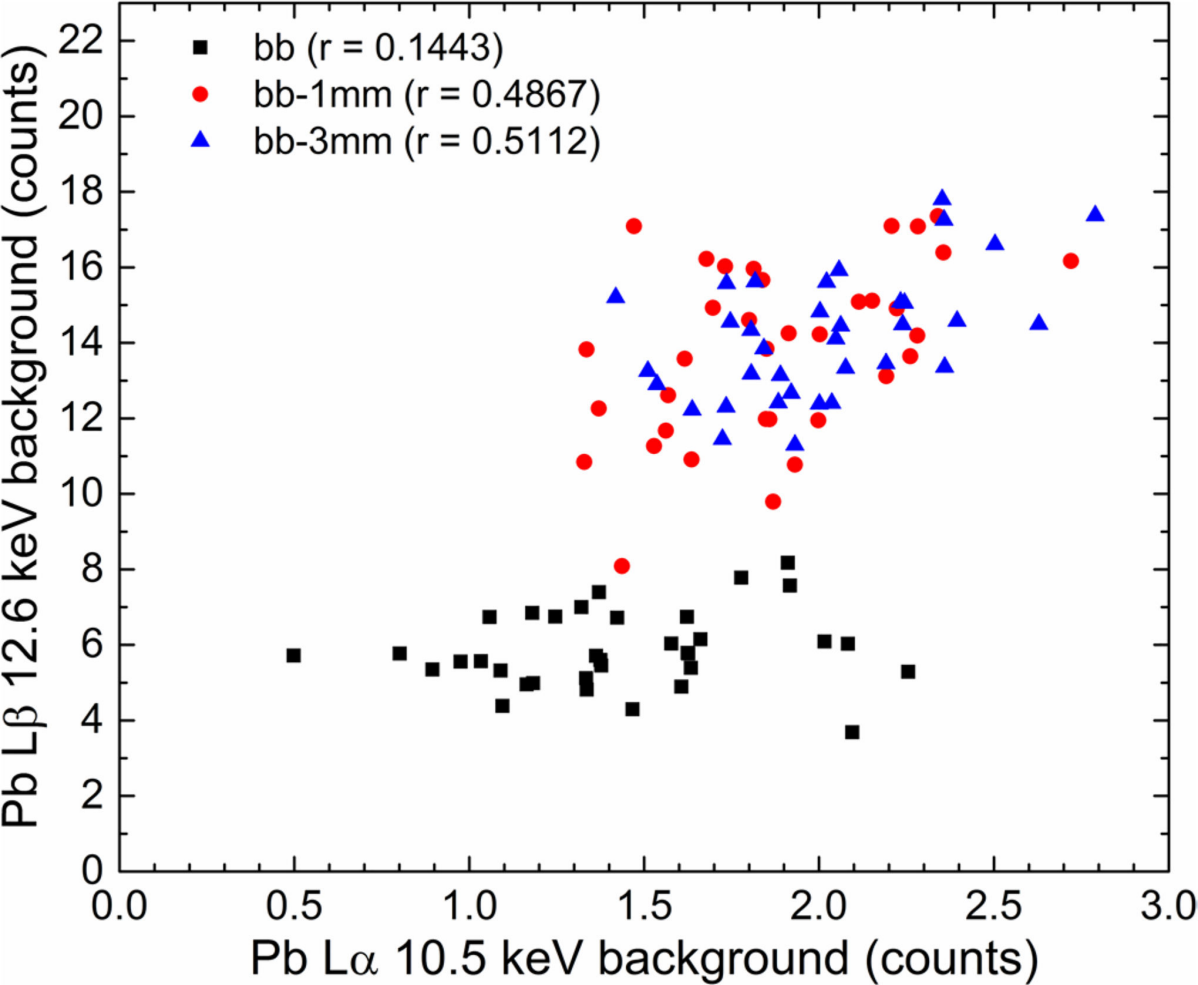
Scatter plot of the fitted background underneath the Pb L*α* and Pb L*β* peaks as calculated at the 10.5 keV and 12.6 keV energy, respectively.

Figure 9 is a scatter plot of the background underneath the Pb L*α* and Pb L*β* peaks calculated at the 10.5 keV and 12.6 keV energies. For each phantom, there were 35 data points corresponding to the five trials for each of the seven Pb-doped bone poP bone phantoms. The Pearson’s correlation coefficient is also provided in the plot’s legend. Substantial overlap between the bb-1 mm and bb-3 mm background data of both peaks can be observed. The difference between the average values of the two data sets was not statistically significant. In other words, the background in the 10–13 keV energy range was essentially the same for the bb-1 mm and bb-3 mm phantoms.

This is a positive outcome of this study and evidence of the scatter reduction due to the employed microbeam grazing-incidence approach. There is, however, clear separation between the bb data (black points) and the bb-1 mm and bb-3 mm data (red and blue data points) for the background underneath the Pb L*β* peak located at the 12.6 keV energy. This increase is due to a larger number of Compton-scattered photons within the POM layer that reached the detector. The incident x-ray beam spectrum from figure 6(d) shows a significant increase in the number of photons for energies above 11 keV. Filtration by the added 1.8 mm Al filter was clearly not sufficient. Additional Al filtration, perhaps at the output-end of the PXL, will have to be investigated in future studies using this microbeam system. An alternative option in background reduction is the use of a confocal XRF method. In this approach, a secondary PXL (Malzer and Kanngießer 2005) or collimating channel array (CCA) (Choudhury *et al* 2017) would guide the x-ray photons emergent from a small targeted volume to the x-ray detector. The limited photon pathways allowed by the secondary PXL would effectively enhance the signal-to-background ratio in the x-ray spectrum even when PXL transmission is taken into account.

### 4.2. Literature comparisons

The DL values of Pb are about two times better than the reported values of Fleming *et al* (2011) who used a portable XRF spectrometer, 1.2 and 2.7 mm thickness of resin as soft tissue phantom material, and the same acquisition times and DL definition. Table 7 summarizes the calibration data from the paper of Fleming *et al* (2011) in which the count rate results were converted to number of counts by multiplying them with their corresponding 180 s acquisition time.

Comparing table 7 values with the current study ones provided in tables 3 and 4, one can notice that the slope values from Fleming *et al* are 5–10 times larger than the current study, while the corresponding uncertainties on the null Pb concentration measurements are more than one order of magnitude larger than the current study. This is perhaps an indicator that the gain in the lower DL values from table 4 could be related to lower scatter background levels in the energy region of the observed Pb LXRF peaks. The portable spectrometer had only a 20 *µ*A current x-ray tube which was 2% of the 1 mA current used in the current study. A direct side-by-side comparison becomes even more complicated when the 10 cm long PXL microbeam collimation and transmission properties and the unknown detector capabilities of the portable spectrometer from Fleming *et al* (2011) study are taken into account.

A Pb DL value of 8.4 *µ*g g^−1^ was reported by Nie *et al* (2011) for 120 s LXRF measurements on poP Pb-doped phantoms with an overlying 2 mm Lucite soft tissue phantom. In their study, the bone and soft tissue phantoms as well as cadaver tibia bones were used to test a novel calibration method and detection limit definition which did not include the traditional peak fitting routine. Therefore, a direct measurement sensitivity comparison is, again, difficult. For a 2 mm POM layer a Pb L*α* calibration line slope *s_α_* (2mm) = 0.042 counts *µ*g^−1^ g can be calculated using the corresponding function and data from table 6. Combined with a *σ*_0_ = 0.2 counts (similar to the values from table 4), a 9.5 *µ*g g^−1^ DL estimate was calculated using equation (4) definition.

### 4.3. *In vivo* Pb concentration LXRF measurement considerations

Several considerations regarding the feasibility of *in vivo* Pb LXRF measurements can be made. The Pb LXRF measurement method presented here used the relatively large Sr K*α* peak to find the optimal grazing-incidence geometry and in systematic uncertainty corrections. A direct translation of the measurement method presented in this study to *in vivo* bone Pb applications poses certain difficulties. It is known that Sr concentration in unexposed human bone ranges in the 0.1–0.3 mg Sr/g Ca (Pejović-Milić 2004). The Sr level in the poP bone phantoms used in the current investigation was unknown. Pejović-Milić *et al* (2004) determined that a commercial poP sample contained about 4 mg Sr/g Ca, approximately an order of magnitude higher than the expected human bone Sr concentration. Therefore, direct implementation would require proportionally longer times which would increase the overall radiation dose cost.

**Figure 10:**
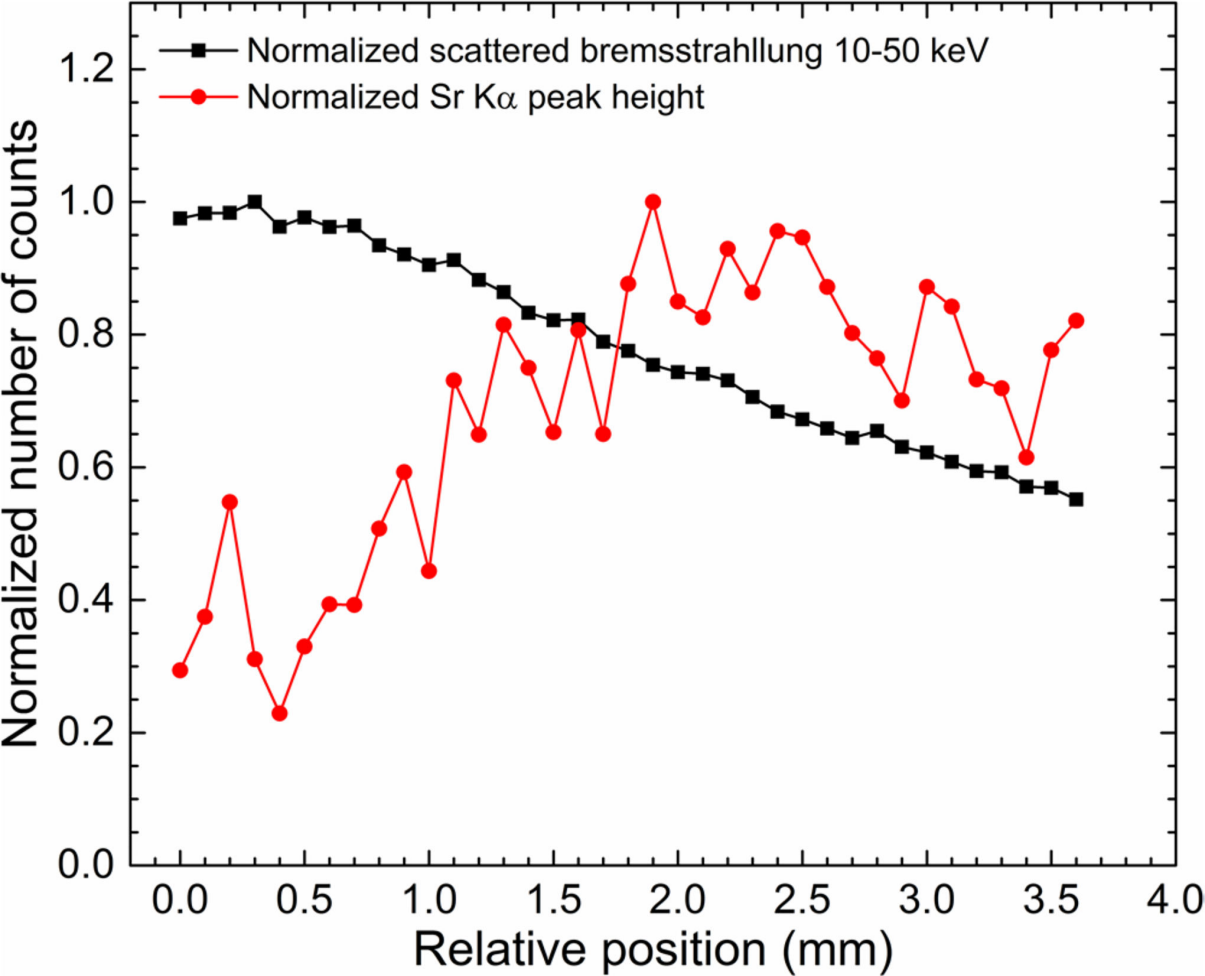
Plots of the normalized (i) sum of the scattered bremsstrahlung photons in the 10–50 keV energy (black line and data points), and (ii) fitted peak height of the Sr K*α* peak (red line and data points) versus the relative position of the bb-3 mm phantom. The corresponding 30 s x-ray spectra also generated data shown in figure 4(a). Normalization was done by dividing the values in each data set by its corresponding maximum value.

The scattered bremsstrahlung peak does not offer, at least in a brief superficial analysis, additional useful information. Figure 10 shows the normalized number of counts in the 10–50 keV scattered bremsstrahlung energy region and the normalized fitted Sr K*α* peak height as a function of the relative position of the bb-3 mm phantom. While the Sr K*α* peak height data plot indicates the optimal grazing-incidence position of the microbeam, the sum of the bremsstrahlung counts values decrease monotonically with the relative position and the curve does not indicate the position for which the microbeam is incident on the bone (at grazing-incidence or not). Alternatively, the grazing-incidence geometry could be determined by a separate soft tissue measurement procedure such as ultrasound (Pejović-Milić *et al* 2002) as done in the past LXRF studies. This approach, however, does not eliminate the issue of intrinsic variations of the x-ray linear attenuation coefficients of the human soft tissues.

The calculated absorbed radiation dose to the POM material corresponding to the 180 s trials was less than 1 mGy. There were unavoidable counting losses in the detection process (dead time was ~1%) and the dose absorbed in the poP bone phantom was neglected. An important approximation overestimating the calculated dose was the radiation absorption volume being taken as equal to the volume along the x-ray beam path in the POM material which was estimated using the lateral beam size measurements in air. The 1 mGy order of magnitude of the radiation dose is the same as reported in the early KXRF study of Somervaille *et al* (1985), but lower than the 10 mGy dose to the skin reported in the pioneering LXRF study of Wielopolski *et al* (1983). As discussed earlier, an added radiation cost was the finding of the optimal grazing-incidence geometry. However, due to the smaller radiation-exposed volumes, six spectral acquisitions of 30 s duration delivered a lower dose to the phantoms than the five 180 s trials used in the Pb LXRF measurements.

An important part of the LXRF Pb concentration measurement applicability to *in vivo* human studies is the calibration method. The early calibration method proposed by Wielopolski *et al* (1983, 1989) was based on ultrasound measurements of soft tissue thickness overlying the bone.

**Figure 11:**
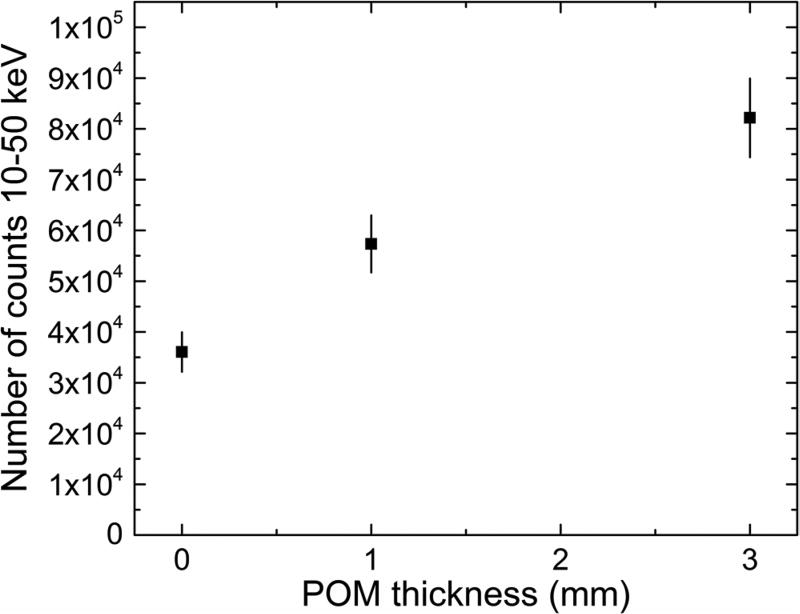
The number of counts in the 10–50 keV energy region of the acquired x-ray spectra. The values and corresponding error bars were calculated as the mean and standard deviation of the mean of the 35 data points for each phantom set.

After more than three decades of research, a novel calibration method is needed to improve the precision and accuracy of Pb LXRF measurements to the levels required by *in vivo* studies. A first step was taken by Nie *et al* (2011) who developed a novel calibration method based on the increase of the observed Compton peak with the overlying soft tissue thickness. Nie *et al* also replaced the traditional peak fitting and calibration line method used in Pb KXRF measurements with a self-titled ‘background subtraction’ method. In it, the authors implicitly assumed that the net increase in the number of counts under the Pb peaks, after the null Pb concentration background is subtracted, is due to the presence of the Pb in the analyzed sample even when the Pb peaks themselves were not resolved. The assumption is not necessarily wrong. A criticism which can be brought forward is that the authors did not provide the reference for a similar precedent in the past or current XRF literature. This data analysis procedure was not tested in this work, but, perhaps, it deserves a closer look. The expected increase in the number of scattered bremsstrahlung photons with increasing thickness of the soft tissue phantom layer was also noticed in this study as shown in figure 11. A similar trend, albeit with more data points and reversed axes, was also found by Nie *et al* (2011). The use of such data to correct the *in vivo* Pb LXRF data for the soft tissue x-ray attenuation is appealing, but requires a careful investigation of all uncertainties involved in such approach.

A different soft tissue x-ray attenuation correction method can be implemented using the grazing-incidence approach proposed in this study. Instead of using the Sr K*α* peak to achieve the optimal grazing-incidence geometry, the microbeam could scan the soft tissue from the top to the location of the bone with the x-ray detector opposite to the PXL. The setup would resemble a traditional x-ray projection scanning imaging method. The scanning distance would provide the soft tissue thickness in the perpendicular direction to the microbeam. The average linear x-ray attenuation coefficient of the soft tissue could be calculated from these measurements provided that the soft tissue thickness in the microbeam direction could be measured by mechanical means such as the use of thin plastic parallel plates. This could mitigate the negative effects of the assumptions regarding x-ray attenuation properties of the soft tissue employed in earlier LXRF studies and discussed by Todd (2002a). At this stage, this proposal is speculative and its radiation dose and additional details can be determined in future LXRF experiments employing this method.

## 5. Conclusions

A table-top microbeam unit was used to implement a grazing-incidence approach to Pb LXRF measurements in poP bone and POM soft tissue phantoms. The calculated Pb DL values based on the calibration line LXRF measurements were in the range 3–40 *µ*g g^−1^ for the 0–3 mm POM thickness range. The radiation dose in the POM material for the 180 s irradiation was estimated to be lower than 1 mGy. The data analysis also demonstrated that the backscatter background corresponding to the observed Pb L*α* and Pb L*β* peaks did not increase with increasing POM soft tissue phantom thickness and was estimated to be a direct effect of the proposed method. The discussion of the methodology and results indicated that a direct implementation of this approach to *in vivo* measurements would be difficult, but viable solutions and ideas using a microbeam were proposed to motivate and encourage future bone Pb LXRF investigations.

## Figures and Tables

**Table 1:** Table of phantom materials and their relevant properties.

Material	Chemical formula	Density (g cm^−3^)
Plaster-of-Paris (poP)	${\text{CaSO}}_{4}\cdot \frac{1}{2}H_{2}O$	1.05 ± 0.05
Polyoxymethylene (POM)	(CH_2_O)_*n*_	1.42 ± 0.14

**Table 2:** Notations of the three sets of Pb LXRF measurements.

Pb LXRF experiment	Notation
Bare bone (poP)	bb
Bare bone and 1 mm thickness cylindrical soft tissue (POM) phantom assembly	bb-1 mm
Bare bone and 3 mm thickness cylindrical soft tissue (POM) phantom assembly	bb-3 mm

**Table 3:** Results of the linear fitting of the raw and corrected Pb L*α* and Pb L*β* peak height data. The numbers in the round parentheses represent the uncertainties in the last significant figure of the corresponding value.

Experiment	Pb L*α*				Pb L*β*			
	*s* (*µ*g^−1^ g)	*y*_0_ (counts)	*χ*^2^/*n*	*p*	*s* (*µ*g^−^1 g)	*y*_0_ (counts)	*χ*^2^/*n*	*p*
bb	0.28(1)	1.2(3)	2.0	>0.05	0.24(2)	0.6(5)	4.1	<0.05
bb-1 mm	0.084(5)	0.3(2)	1.4	>0.05	0.10(2)	−0.2(5)	2.3	<0.05
bb-3 mm	0.026(5)	0.6(2)	1.8	>0.05	0.030(4)	0.07(1)	0.9	>0.05

	Corrected Pb L*α*				Corrected Pb L*β*			

bb	0.322(8)	0.8(2)	0.5	>0.05	0.28(2)	0.7(4)	2.0	>0.05
bb-1 mm	0.087(5)	0.5(2)	1.2	>0.05	0.11(2)	0.3(5)	1.1	>0.05
bb-3 mm	0.024(5)	0.7(2)	1.5	>0.05	0.030(4)	0.1(1)	0.7	>0.05

**Table 4:** Null Pb concentration uncertainties *σ*_0_ and calculated DL results. The numbers in the round parentheses represent the uncertainties in the last significant figure of the corresponding value.

	Corrected Pb L*α*		Corrected Pb L*β*	
Experiment	*σ*_0_ (counts)	DL (*µ*g g^−1^)	*σ*_0_ (counts)	DL (*µ*g g^−1^)
bb	0.3	3.1(1)	0.3	2.9(2)
bb-1 mm	0.1	4.9(3)	0.6	18(3)
bb-3 mm	0.3	39(8)	0.2	23(3)

**Table 5:** X-ray linear attenuation coefficients of the phantom materials for relevant x-ray photon energies.

Photon energy (keV)	10.5	12.6	15.2
Atomic transition	Pb L*α*	Pb L*β*	Pb L_3_ edge
*µ*_poP_ (mm^−1^)	3.15	1.88	1.10
*µ*_POM_ (mm^−1^)	0.511	0.303	0.181

**Table 6:** Results of the calibration line slope data fitting with the x-ray attenuation model.

Slope data	Fitting function: *s* = *s* (*t*)	*s_α,β_* (0) (counts *µ*g^−1^ g)	Parameter *a* (mm^−1^)	*χ*^2^/*n*
Pb L_*α*_	$s_{\alpha }(t)=s_{\alpha }(0)\text{exp}[-\text{at}-0.181\cdot {\left(30t+t^{2}\right)}^{\frac{1}{2}}]$	0.322	0.29 ± 0.01	0.11
Pb L_*β*_	$s_{\beta }(t)=s_{\beta }(0)\text{exp}[-\text{at}-0.181\cdot {\left(30t+t^{2}\right)}^{\frac{1}{2}}]$	0.28	0.12 ± 0.03	0.71

**Table 7:** Calibration data summary from Fleming *et al* (2011).

	Pb L*α*		Pb L*β*	
Phantom	*σ*_0_ (counts)	Slope (counts · *µ*g^−1^g)	*σ*_0_ (counts)	Slope (counts · *µ*g^−1^g)
bb-circular	4.3	1.08 ± 0.04	3.6	1.13 ± 0.04
bb-square	4.1	0.97 ± 0.05	4.0	1.0 ± 0.05
bb-square and 1.2 mm resin	4.7	0.58 ± 0.07	5.0	0.61 ± 0.07
bb-circular and 2.7 mm resin	7.9	0.25 ± 0.04	6.3	0.40 ± 0.04
